# Persistent Müllerian Duct Syndrome with Transverse Testicular Ectopia: A Novel Anti-Müllerian Hormone Receptor Mutation

**DOI:** 10.4274/jcrpe.4058

**Published:** 2017-06-01

**Authors:** Özlem Korkmaz, Samim Özen, Nurhan Özcan, Petek Bayındır, Sait Şen, Hüseyin Onay, Damla Gökşen, Ali Avanoğlu, Ferda Özkınay, Şükran Darcan

**Affiliations:** 1 Ege University Faculty of Medicine, Department of Pediatric Endocrinology, İzmir, Turkey; 2 Ege University Faculty of Medicine, Department of Radiology, İzmir, Turkey; 3 Ege University Faculty of Medicine, Department of Pathology, İzmir, Turkey; 4 Ege University Faculty of Medicine, Department of Genetics, İzmir, Turkey; 5 Ege University Faculty of Medicine, Department of Pediatric Surgery, İzmir, Turkey

**Keywords:** Undescended testis, anti-Müllerian hormone receptor mutation, anti-Müllerian hormone receptor resistance

## Abstract

Persistent Müllerian duct syndrome is the result of either anti-Müllerian hormone (AMH) deficiency or AMH receptor resistance. A long tubular structure was palpated during the physical examination of a 13-month-old male patient who had presented with bilateral undescended testes. At physical examination, the testes were not palpable. The patient’s karyotype was XY, SRY (+), and his AMH level was 22 ng/mol. Structures suggestive of ovaries, a uterus, and fallopian tubes were observed during the laparoscopic examination of the ectopic testis. *AMHR2* gene sequence analysis performed with a preliminary diagnosis of AMH receptor resistance revealed a previously unreported homozygous c.24G>A (p.W8X) mutation. The patient was assessed as a case of AMH receptor resistance. Orchiopexy was performed.

## What is already known on this topic?

Persistent Müllerian duct syndrome (PMDS) is a disorder of sexual differentiation characterised by the persistence of Müllerian derivatives in males with an XY karyotype and normal virilization. PMDS are caused by mutations in the anti-Müllerian hormone gene, which lead to defects in its secretion or activity, or to mutations in the gene for the type II receptor for anti-Müllerian hormone, which results in a clinical picture of hormonal resistance.

## What this study adds?

We report a previously undescribed homozygous c.24G>A (p.W8X) mutation determined at AMHR2 gene analysis.

## INTRODUCTION

Persistent Müllerian duct syndrome (PMDS) is a rare disorder of 46,XY sex development. It occurs as a result of anti-Müllerian hormone (AMH) deficiency or AMH receptor resistance, conditions which arise due to mutations in the AMH gene or the *AMH type 2 receptor (AMHR2)* gene. In these patients, the external genital structure is that of a normal virilized male, while fallopian tubes and a uterus are observed in the internal genital structure (1,2). Here, we report a case of PMDS in a 13-month-old male presenting with bilateral cryptorchidism and a novel homozygous mutation in the *AMHR2* gene.

## CASE REPORT

A 13-month-old male presented to the pediatric surgery department with a complaint of bilateral undescended testis. A long, tubular structure (testis?) was palpated within the canal. Ultrasonography revealed absence of a testicular structure in the left inguinal canal or scrotum. On the right side, two structures measuring 12x8 mm and 11x7 mm and thought to be testes were observed in the proximal and middle parts of the inguinal canal. These findings were interpreted as transverse testicular ectopia on the right. During laparoscopic examination of the ectopic testis, tissues suggestive of a uterus, fallopian structures, and ovaries were observed inside the abdomen. Gonad biopsy was performed and the patient was referred to the pediatric endocrinology department with a preliminary diagnosis of sexual development disorder. At physical examination, weight was 10.2 kg [standard deviation score (SDS): -1.06] and height was 81 cm (SDS: -0.39). Blood pressure was 98/60 mm/Hg, Quickly score 2. The testes were not palpable and the phallus was 3.5 cm in length. The mother and father were first-degree relatives. Karyotype analysis revealed a XY, SRY (+) karyotype. Serum follicle-stimulating hormone level was 0.44 mIU/L (0.26-3 mIU/L), luteinizing hormone was 0.27 mIU/L (0.02-0.3 mIU/L), free testosterone 0.3 pg/mL (0.15-0.6 pg/mL), total testosterone 0.1 ng/dL (0.2-1.3 ng/dL), E_2_ <20 ng/mL (<15 ng/mL), and AMH >22 ng/mL (4.9-264.5 ng/mL). Immature seminiferous tubular structures were observed in gonad biopsy specimens sent from the pediatric surgery department. *AMHR2* gene sequence analysis performed with a preliminary diagnosis of AMH receptor resistance revealed a previously undescribed homozygous c.24G>A (p.W8X) mutation. The parents had the same mutation in heterozygous form. The patient was evaluated as a case of AMH receptor resistance and presented to our “Sex Development Disorders Council”. Upon their decision, orchiopexy was performed.

## DISCUSSION

AMH is secreted from immature Sertoli cells in males and from ovarian granulosa cells in females and is responsible for the regression of the Müllerian ducts in the male fetus. The clinical picture that appears in the 46,XY genotype in AMH synthesis and effect deficiency is known as PMDS. This occurs as a result of mutations in the *AMH* gene or in the *AMHR2* gene. Serum AMH levels are low or undetectable in *AMH* gene mutations, while they are normal or elevated in *AMHR2* gene mutations (1,2).

Most cases of PMDS are diagnosed following a virilized XY patient presenting with bilateral or unilateral undescended testis or inguinal hernia. Three anatomic variants of PMDS have been described. In group 1, bilateral testicles are located intraabdominally. In group 2, one testis is located in a hernia sac or along with the Müllerian structures (hernial uterus inguinalis). In group 3, both testes are found in the same hernia sac, along with uterus and tubes (crossed or transverse testicular ectopia) (3,4). Given that the genotype of AMH and AMHR2 is not related to the phenotypes, the phenotype of our index case was consistent with group 3 (5,6). Our patient presented to the Pediatric Surgery Department with a complaint of bilateral undescended testes and was referred to us when female internal genital structures were observed at laparoscopy. It has been reported that the undescended testes in PMDS may undergo malignant transformation. The prevalence of germ cell tumors such as seminoma, most commonly, as well as embryonal carcinoma, yolk sac tumor and, more rarely, clear cell adenocarcinoma in such patients varies from 15-40%, a frequency no higher than that reported for abdominal testes in general (7,8). Orchiopexy is therefore recommended as early as possible in these cases (9). In our case, too, orchiopexy was performed on both testes seen on the same side inside the abdomen, and both right and left were enabled to descend into the scrotum.

AMH is a member of the TGF-beta family. In males, serum levels of AMH remain high until 2 years of age and persist in measurable levels until puberty, before decreasing to undetectable levels at puberty (10,11). Low or undetectable AMH levels in cases with PMDS indicate AMH mutation, whereas high AMH levels may indicate mutations in the *AMHR2* gene (12). The *AMH* gene is located on the short arm of the 19^th^ chromosome and was first cloned in 1986 by Cate et al (13). It consists of five exons and is 2.8 kbp in length. PMDS exhibits an autosomal recessive pattern. Mutation in the *AMH* gene or *AMHR2* gene has been reported in 84% of cases. *AMHR2* gene mutations are located in the long arm 13.13 (12q.13.13) region of the 12^th^ chromosome. *AMHR2* contains 11 exons and more than 27 mutations have already been described in this gene (14,15). The *AMHR2* gene contains four intronic polymorphisms, located in t276a intron 1, c1280t intron 3, c1827t intron 5, and a6503g intron 10 (16). In a study of 32 families with PMDS, Imbeaud et al (17) determined mutations in the *AMH* gene in 16 families and in the *AMHR2* gene in the other 16. Deletion 27-bp in size was observed in the 10^th^ exon in 10 of the 16 patients with mutation in the *AMHH2* gene. This mutation has been reported as the cause of 25% of cases of PMDS (17). AMH receptor resistance was primarily considered in our case due to AMH level. At mutation analysis, a previously undefined homozygous c.24G>A (p.W8X) mutation was determined at *AMHR2* gene analysis. Mutation screening revealed that the mother and father bore the same mutation in heterozygous form.

In conclusion, in cases with bilateral cryptorchidism, the clinicians should be aware of the possibility of PMDS. The condition should be considered when persistent Müllerian structures are observed, particularly in virilized males with a normal external genital structure.
